# Lichen acclimation to changing environments: Photobiont switching vs. climate‐specific uniqueness in *Psora decipiens*

**DOI:** 10.1002/ece3.2809

**Published:** 2017-03-16

**Authors:** Laura Williams, Claudia Colesie, Anna Ullmann, Martin Westberg, Mats Wedin, Burkhard Büdel

**Affiliations:** ^1^ Plant Ecology and SystematicsBiology InstituteUniversity of KaiserslauternKaiserslauternGermany; ^2^ Museum of EvolutionUppsala UniversityUppsalaSweden; ^3^ Department of BotanySwedish Museum of Natural HistoryStockholmSweden

**Keywords:** biological soil crusts, environmental change, Europe, genetic diversity, green algae, latitudinal gradient, morphological variability, *Myrmecia*, plant–climate interactions, plasticity

## Abstract

Unraveling the complex relationship between lichen fungal and algal partners has been crucial in understanding lichen dispersal capacity, evolutionary processes, and responses in the face of environmental change. However, lichen symbiosis remains enigmatic, including the ability of a single fungal partner to associate with various algal partners. *Psora decipiens* is a characteristic lichen of biological soil crusts (BSCs), across semi‐arid, temperate, and alpine biomes, which are particularly susceptible to habitat loss and climate change. The high levels of morphological variation found across the range of *Psora decipiens* may contribute to its ability to withstand environmental change. To investigate *Psora decipiens* acclimation potential, individuals were transplanted between four climatically distinct sites across a European latitudinal gradient for 2 years. The effect of treatment was investigated through a morphological examination using light and SEM microscopy; 26S rDNA and rbcL gene analysis assessed site‐specific relationships and lichen acclimation through photobiont switching. Initial analysis revealed that many samples had lost their algal layers. Although new growth was often determined, the algae were frequently found to have died without evidence of a new photobiont being incorporated into the thallus. Mycobiont analysis investigated diversity and determined that new growth was a part of the transplant, thus, revealing that four distinct fungal clades, closely linked to site, exist. Additionally, *P. decipiens* was found to associate with the green algal genus *Myrmecia,* with only two genetically distinct clades between the four sites. Our investigation has suggested that *P. decipiens* cannot acclimate to the substantial climatic variability across its environmental range. Additionally, the different geographical areas are home to genetically distinct and unique populations. The variation found within the genotypic and morpho‐physiological traits of *P. decipiens* appears to have a climatic determinant, but this is not always reflected by the algal partner. Although photobiont switching occurs on an evolutionary scale, there is little evidence to suggest an active environmentally induced response. These results suggest that this species, and therefore, other lichen species, and BSC ecosystems themselves may be significantly vulnerable to climate change and habitat loss.

## 1 Introduction

Biological Soil Crusts (BSCs) are biologically modified soil surfaces composed of an amalgamation of organisms which include lichens, bryophytes, microalgae, (cyano) bacteria, and microfungi. They are often the dominant vegetation type in areas limited by either water availability or temperature, and provide vital ecosystem services such as soil stabilization and nutrient acquisition (Belnap, 2003). Investigating BSCs has gained significant interest in recent years as their global importance has become highlighted. Many studies aim to increase knowledge on diversity in different biomes (Büdel et al., 2009; Pushkareva & Elster, 2013; Rosentreter, Eldridge, Westberg, & Williams, 2016; Seppelt, Downing, Deane‐Coe, Zhang, & Zhang, 2016; Williams, Loewen‐Schneider, Maier, & Büdel, 2016), and others investigate the roles BSCs play in ecosystem function (Colesie, Green, Haferkamp, & Büdel, 2014; Elbert et al., 2012; Keck, Felde, Drahorad, & Felix‐Henningsen, 2016; Pietrasiak et al., 2013). BSCs are complex communities due to the many different organisms involved and functional aspects they provide (Belnap et al., 2001). However, a major proportion is often comprised of lichens, which constitutes a climax stage in BSC development worldwide. *Psora decipiens* (Hedw.) Hoffm. is one such contributing lichen, being a generalist species found to dominate in climatically distinct BSC regions around the world (Büdel, 2003; Galun & Garty, 2003; Rosentreter & Belnap, 2003; Timdal, 2010). Regardless of its worldwide distribution, research on this important lichen species has been minimal.

Lichens are a symbiotic relationship between fungal and algal (photobiont) partners, allowing colonization of habitats where the individual organism could not survive. Numerous studies have investigated the relationships between the fungi and its photobiont (e.g., Dal Grande et al., 2014; Fernández‐Mendoza et al., 2011; Kroken & Taylor, 2000; O'Brien, Miadlikowska, & Lutzoni, 2005; Piercey‐Normore, 2004) and many lichen families, genera, and species have been shown to associate with an array of algal partners (e.g., Beck, Kasalicky, & Rambold, 2002; Muggia, Baloch, Stabenteiner, Grube, & Wedin, 2011; Muggia et al., 2013; Nyati, Scherrer, Werth, & Honegger, 2014; O'Brien, Miadlikowska, & Lutzoni, 2013; Romeike, Friedl, Helms, & Ott, 2002; Thüs et al., 2011). Although never conclusively shown, this can be assumed to allow the lichen to adapt to different environments (Blaha, Baloch, & Grube, 2006; Yahr, Vilgalys, & Depriest, 2006) and may allow a widening of their ecological niche. Photobiont switching is the mechanism which allows a specific lichen fungus to associate with a new algal partner and has been shown to occur throughout lichen evolution (Henskens, Green, & Wilkins, 2012; Magain & Sérusiaux, 2014; Muggia, Grube, & Tretiach, 2008; Nelsen & Gargas, 2008; Piercey‐Normore & Depriest, 2001). However, many questions around photobiont switching are unanswered, how a lichen selects an algal partner is unknown, whether a lichen can actively choose a photobiont from a local pool remains unclear, and nothing is known about time scales over which photobiont switching can occur. Being able to switch photobionts actively would allow lichens to acclimate to changing environmental conditions, presumably by selecting an algal partner that is specifically adapted to those conditions. Acclimation refers to the ability of an organism to modify its gene expression, and hence, physio‐morphological features, in response to the environment. This is in contrast to adaption, which refers to actual changes in an organism's genome (Giordano, 2013). To some extent, the ability of lichens to acclimate to their environment has been of interest to lichenologists for many years. Larson and Kershaw (1975) found evidence for acclimation in arctic lichens, discovering rapid acclimation to temperature, light and thallus moisture content. In more recent years, lichens have been found to acclimate their respiration in response to seasonal temperatures (Lange & Green, 2005), and transplants were found to acclimate to high light by increasing thallus thickness and chlorophyll a/b‐ratio (Gauslaa, Lie, Solhaug, & Ohlson, 2006). A closely related topic discusses phenotypic plasticity in lichens: the ability of a genotype to develop various phenotypes in response to different environmental conditions (Vallardes, Gianoli, & Gómez, 2007). Many lichen species have been shown to have differing ecophysiological and morphological traits dependent on the ecological niche inhabited (e.g., Muggia, Pérez‐Ortega, Fryday, Spribille, & Grube, 2014; Pérez‐Ortega et al., 2012; Pintado, Valladares, & Sancho, 1997; Printzen, Domaschke, Fernández‐Mendoza, & Pérez‐Ortega, 2013; Tretiach & Brown, 1995). Phenotypic plasticity and the ability to actively acclimate to environmental conditions would allow species to withstand pressure from climate change, human disturbance, and habitat loss.

Recently, two climatically distinct populations of *P. decipiens* were studied in order to assess whether ecophysiological and/or morphological traits could explain the ability to thrive in diverse habitats (C. Colesie , L. Williams, & B. Büdel, submitted). The results suggested that the regulation of thallus water content allowed individuals to be specifically adapted to conditions in a semi‐arid region compared to those of a wet, alpine region. This contribution intends to explore whether genetically fixed adaption or acclimation is responsible for this variability. By installing a transplant experiment between climatically variable sites in western Europe, the ability of *P. decipiens* to acclimate across its range can be investigated. It is expected that the transplanted lichens acclimate to the new environment by associating with a locally adapted photobiont and by modifying their morpho‐physiological traits. Currently, *Asterochloris* (Schaper & Ott, 2003) and/or *Trebouxia* species are thought to be the photobionts of *P. decipiens*, with high diversity within and between populations (Ruprecht, Brunauer, & Türk, 2014). Climatic factors have been shown to be significant determinants of *Asterochloris* lineages associated with lichens of the genera *Lepraria* and *Stereocaulon* (Peksa & Skaloud, 2011). Therefore, we suggest that if a *P. decipiens* transplant can acclimate, a new photobiont will be required.

## 2 Materials and Methods

### 2.1 Study sites

In order to cover a broad range of different macro‐climatic conditions, four study sites were selected across Europe. The four sites in this study have previously been described in full (Büdel et al., 2014; Williams et al., 2016), and therefore, the following provides only a brief introduction. All climate data were obtained from weather stations installed at each site and covers a period of 2 years (2012–2014).

1. Sweden—Nature Reserve Gynge Alvar, Öland (56°32′N, 16°28′E). The site is situated ca. 20 m a.s.l and has a maritime climate, with roughly 500 mm annual precipitation and an average temperature of 8°C.
2. Germany—Nature Reserve “Ruine Homburg,” Gössenheim, northern Bavaria (50°01′N, 9°48′E). The site lies at 295 m a.s.l and has a warm temperate climate with average temperatures of 9.5°C. Annual precipitation is 600 mm.
3. Austria—Hochtor, Hohe Tauern National Park (47°05′N, 12°51′E). Situated at ca. 2,600 m a.s.l, the site is located near the Großglockner High Alpine Road. Annual precipitation is between 1,750 mm and 2,000 mm of which 70% falls as snow and the mean annual temperature is −1°C.
4. Spain—Tabernas Badlands, “Paraje Natural” Almeria (37°00′N, 2°26′W). This semi‐arid warm‐Mediterranean site has an altitude of 250 m a.s.l and only 220 mm of precipitation annually which on average falls across 37 days. The mean annual temperature is 18.5°C.

Spain and Austria are sites where BSC occurs naturally due to the environmental conditions; in comparison, the Swedish and German sites are, at least to some extent, maintained through human intervention, such as cattle grazing. Due to the extremities in temperature and precipitation at the natural sites, they are here referred to as the extreme sites compared to the milder, temperate sites.

### 2.2 Transplantation

**Figure 1:**
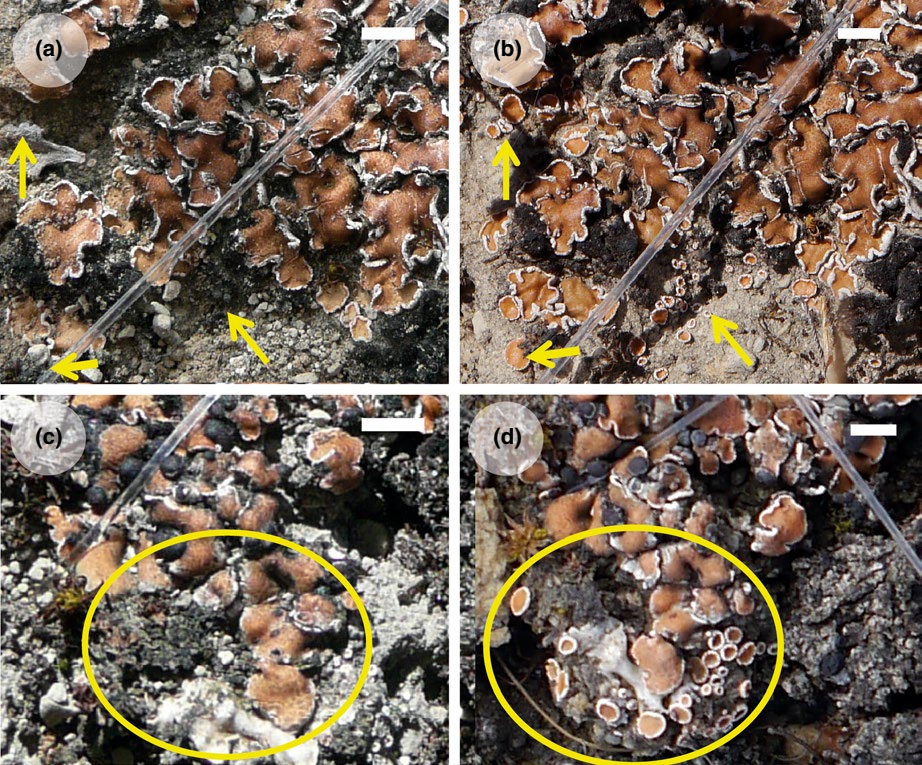
*Psora decipiens* transplants showing new‐grown thalli after 1 year (a) Germany to Spain initially (b) 1 year later (c) Germany to Austria initially (d) 1 year later. Scale = 5 mm

*Psora decipiens* samples were collected from every site in 2012. Each sample was at least a 9 cm^2^ section of intact BSC dominated by multiple *P. decipiens* thalli (Figure 1). Samples were air‐dried at room temperature and stored at −20°C before use. Five replicates from each site were transplanted between all four sites, including a control which entailed transplantation within the site, to test for any effect of the process itself (number of transplant combinations was 15, see Table 1). All samples were installed in the new site within 6 months of collection and remained in the field for 1.5–2 years. All samples were collected between May and August 2014, once again air‐dried, and stored at −20°C before further investigation.

**Table 1:** Results of the transplantation experiment regarding the growth of new thalli. Numbers represent the number of replicates, of five, with new growth identified after transplantation treatment

↓ From	→ To			
	Austria	Sweden	Germany	Spain
Austria	3	1	4	3
Sweden	0	1	3	0
Germany	1	3	0	2
Spain	3	1	2^a^	2

a Fungal phylogeny demonstrates that the new growth did not originate from Spanish sample.

### 2.3 Morphological analysis

Thalli that had grown during transplantation were first identified through photographic comparison and used, where possible, in all investigations (Figure 1). Thallus internal structure was visualized through freezing microtome sections and light microscopy using an Axioskop microscope with AxioVision software (Carl Zeiss, Jena, Germany). For electron microscopy a low‐temperature scanning electron microscope (Supra 55VP; Carl Zeiss, Oberkochen, Germany) was used to study fully hydrated lichen specimens. The samples were frozen in liquid nitrogen slush (K1250X Cryogenic preparation system, Quorum technologies; Ashford, UK) and mounted on special brass trays. After sublimation at −80°C for 30 min, samples was sputter‐coated with gold–palladium and viewed at a temperature of −130°C and 5 kV accelerator voltage.

Biological soil crusts from directly below lichen thalli were cultured on green algal medium (MBB) in order to investigate availability of free‐living green algae for potential photobiont switching. Cultures were maintained in a culture room at 17°C under a 14 hr: 10 hr, light: dark regime at a light intensity of 10–40 μmol photons m^−2^ s^−1^ provided by daylight cool white fluorescent lamps. Algal cells were visualized by light microscopy, as above, after 4–8 weeks, and identified according to Ettl & Gärtner, 1995.

### 2.4 Molecular analysis

Lichen thalli were thoroughly washed to remove any epiphytic fungi, algae, and loose soil. Individual thalli were detached from any remaining soil particles under a binocular microscope. Total genomic DNA was extracted using a CTAB method followed by phenol–chloroform–isoamyl alcohol purification adapted for lichens from Shivji, Rogers, & Stanhope, 1992. Briefly, lichen samples were frozen in liquid nitrogen and ground; 500 μl of Buffer B (1.4 M NaCl, 20 mM EDTA Na_2_, 100 mM Tris‐HCl pH 8.6), 100 μl of CTAB (90°C, 10%), and 50 μg of Proteinase K were added to the sample and shaken in a Thermoblock at 60°C for 1 hr. Chloroform: isoamyl alcohol (24:1) and phenol rothiphenol: chloroform (1:1) solutions were used for purification before precipitation and resuspension in 30 μl of TE buffer; DNA was stored at −20°C. In addition to the controls and transplants from the four sites, two samples from South Africa, one from Tunisia, and one from Portugal were also included to act as comparisons. See Table A1 in Appendix for specimen lists and accession numbers.

Each DNA extraction contained both the fungal and algal symbionts, and general green algal primers were used. Therefore, the results confirm that the obtained sequences come from the intended lichens and not from epiphytic green algae because a contamination would have resulted in a mixed sequence. The algal DNA was amplified using the green algae‐specific forward primer All500af (GCGCGCTACACTGATGC: Helms, Friedl, Rambold, & Mayrhofer, 2001) from the 18s (SSU) rDNA and the reverse, general primer LR3 (CCGTGTTTCAAGACGG: Friedl & Rokitta, 1997) from the 26S rDNA (LSU). Initially, various sequencing primers were tested to target the ITS 1 and 2 gene regions of the rDNA. However, multiple gene copies frequently caused mixed, unusable sequences so an alternative was sought. The 26s rDNA region could be successfully amplified with the LR3 primer for all samples. Friedl & Rokitta, (1997) and Buchheim et al.*,* (2001) have both shown the merits of utilizing this gene in algal species delimitation and was therefore utilized throughout this study. In addition to the 26S rDNA, amplification and sequencing of the large subunit (rbcL) of the plastid gene ribulose‐1, 5‐biphosphate carboxylase/oxygenase was implemented, using the primers rbcL fwd and rbcL rev (Nyati et al., 2014) (rbcL fwd: GAMACTGATATTCTTCTTGCAGC, rbcL rev: GCAGCTAATTCAGGACTCCA). Fungal nITS rDNA was also amplified from the 18s rDNA to the 26s rDNA, using the primers ITS1F (CTTGGTCATTTAGAGGAAGTAA: Gardes & Bruns, 1993) and LR3 (http://sites.biology.duke.edu/fungi/mycolab/primers.htm). *All500af/LR3*: PCR was performed in 50 μl reactions with HotStarTaq^®^ Plus DNA Polymerase Kit (Qiagen, Hilden, Germany) containing 2.25 mM mg^2+^, 200 μm dNTP mix (10 mM of each), 1.25 units of DNA Polymerase, 0.1 μM of each primer (Eurofins MWG, Ebersberg, Germany), and 100 ng DNA. PCR conditions were as follows: Initial denaturization at 95°C for 15 min followed by 35 cycles at 94°C for 45 s, 54°C for 45 s, 72°C for 90 s, with a final extension step of 72°C for 10 min. *RbcL fwd/RbcL rev*: PCR was performed in 25 μl reactions with HotStarTaq^®^ Plus DNA Polymerase Kit (Qiagen, Hilden, Germany) containing 3 mM mg^2+^, 200 μM dNTP mix (10 mM of each), 1.25 units of DNA Polymerase, 0.4 μM of each primer (Eurofins MWG, Ebersberg, Germany), and 100 ng DNA. PCR conditions were as follows: Initial denaturization at 95°C for 15 min followed by 31 cycles at 95°C for 45 s, 52°C for 60 s, 72°C for 80 s, with a final extension step of 72°C for 10 min. *ITS1F/LR3*: PCR was performed in 25 μl reactions with HotStarTaq^®^ Plus DNA Polymerase Kit (Qiagen, Hilden, Germany) containing 1.5 mM mg^2+^, 200 μM dNTP mix (10 mM of each), 1.25 units of DNA Polymerase, 0.2 μM of each primer (Eurofins MWG, Ebersberg, Germany), and 100 ng DNA. A touchdown PCR was performed with the following conditions: Initial denaturization at 95°C for 15 min followed by three cycles of 95°C for 40 s, 58°C for 40 s, 72°C for 90 s, three cycles of 95°C for 40 s, 56°C for 40 s, 72°C for 90 s and 29 cycles of 95°C for 40 s, 54°C for 40 s, 72°C for 90 s, with a final extension step of 72°C for 8 min. All amplicons were processed by SeqIT Kaiserslautern.

Sequence chromatograms were visualized in Sequencher (version 4.5), and sequences were BLASTed against the GenBank database (http://blast.ncbi.nlm.nih.gov/Blast.cgi). *Trebouxia*,* Asterochloris* and *Myrmecia* rbcL sequences were incorporated into the algal analysis, with *Trebouxia* sequences proving to be a suitable outgroup. Sequences were also BLASTed against each other to gauge sequence identity. Sequence matrices were constructed in Seaview (version 4.3.3), the Muscle algorithm (Edgar, 2004) was used for alignment, and manual editing, excluding ambiguous regions and introns, was carried out in MEGA version 6 (Tamura, Stecher, Peterson, Filipski, & Kumar, 2013). Maximum‐likelihood trees with 500 bootstrap replications were inferred using PAUP (version 4.0a149) with GTR model + gamma distribution as recommended by jModelTest (version 0.1.0), which was implemented in the web package Phylemon 2.0 (Sánchez et al., 2011). The South African *Psora decipiens* mycobiont sequences were found to form a separate clade from all other sequences and were therefore designated as the outgroup for the ML trees. Posterior probabilities were calculated through Mr Bayes MCMC analyses (version 3.1.2), and PhyML‐Best‐AIC trees (version 1.02b) were also reconstructed for further comparison. These tests were also implemented in Phylemon 2.0. ML, and Bayesian trees were constructed for all genes individually and for the concatenated sequences of the rbcL and 26S rDNA green algal sequences. The two algal genes were found to produce congruent trees throughout the methodologies with the concatenated sequences generating the highest support. The fungal tree reconstruction was congruent through all utilized methodologies. Trees were graphically displayed with FigTree (version 1.3.1), ML bootstrap support for nodes ≥ 50%, and the posterior probabilities from the Bayesian analysis, node values ≥70%, were incorporated.

## 3 Results

**Figure 2:**
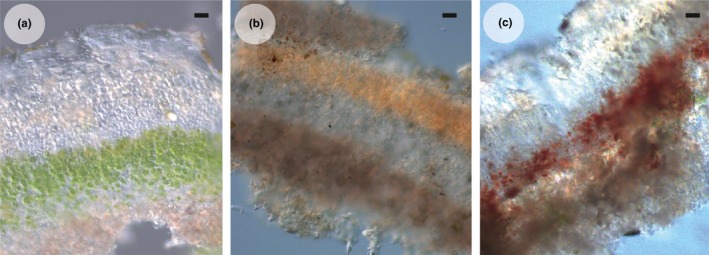
Cross sections of *Psora decipiens* transplants showing a living (a) or dead (b, c) photobiont layer, (a) Spain to Germany (b) Sweden to Spain (c) Spain to Austria. Scale bar = 20 μm

**Figure 3:**
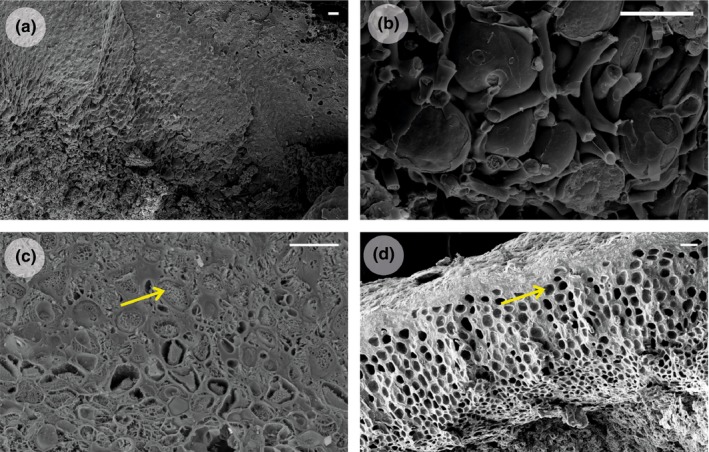
Scanning electron microscope (SEM) photographs of *Psora decipiens* cross sections, (a, b) Spain control showing intact photobiont layer and healthy algal cells, (c) Austria to Spain transplant showing fungal hyphae growing within the algal cell cavity, (d) Spain to Austria transplant showing cavities devoid of algal cell remnants. Scale bar = 10 μm

Initially*, Psora decipiens* samples appeared to not only survive being transplanted to a new environment but to thrive, as can be seen by the substantial new growth in Figure 1 and the numbers of replicates with new growth per transplant combination in Table 1. Therefore, the discovery of thalli transplanted to Austria and Spain without an algal layer once the morphological investigation began was unexpected (Figure 2). Thalli, from each transplanted sample, were examined, and the algal layer was found to be missing in both the transplanted and new‐grown thalli of all replicates that had been transplanted to a site where the climate was more extreme than the original, for example, Sweden (temperate) to Austria (alpine) (Table 2). In three transplant combinations (Sweden to Austria, Sweden to Spain, and Germany to Spain), small pockets of remaining algae were discovered in protected positions and used in downstream investigation (Table 2). Transplantation between sites where the climatic change was not toward either the semi‐arid or alpine sites, for example, Spain to Germany, and the control within site transplants, resulted in new‐grown thalli and living algal layers in both the transplanted and new thalli (Tables 1 and 2, and Figure 2). To investigate further, the disappearance of the photobiont layer SEM was employed. The healthy intact algal cells could easily be discerned in the control samples as seen in Figure 3a,b. However, in the transplants where the photobiont layer had disappeared, the SEM revealed fungal hyphae growing within the algal cell cavities (Figure 3c), or completely empty cell cavities (Figure 3d) without any remaining algal cell material.

**Table 2:**
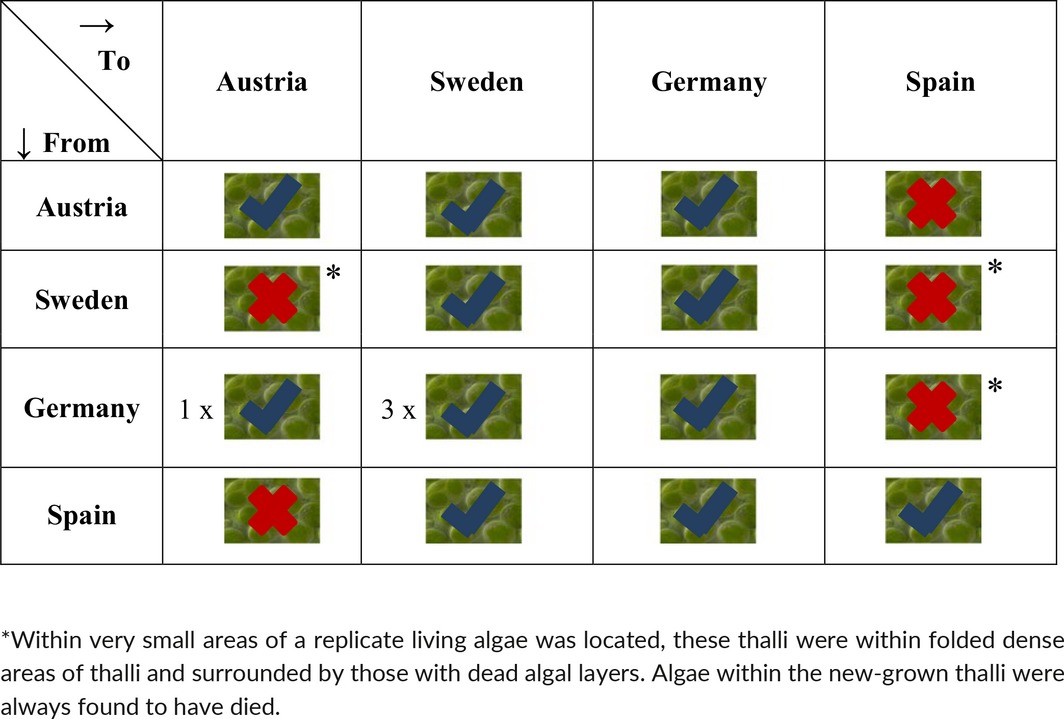
Results of the transplantation experiment regarding whether the photobiont layer remained alive after the transplantation procedure

**Figure 4:**
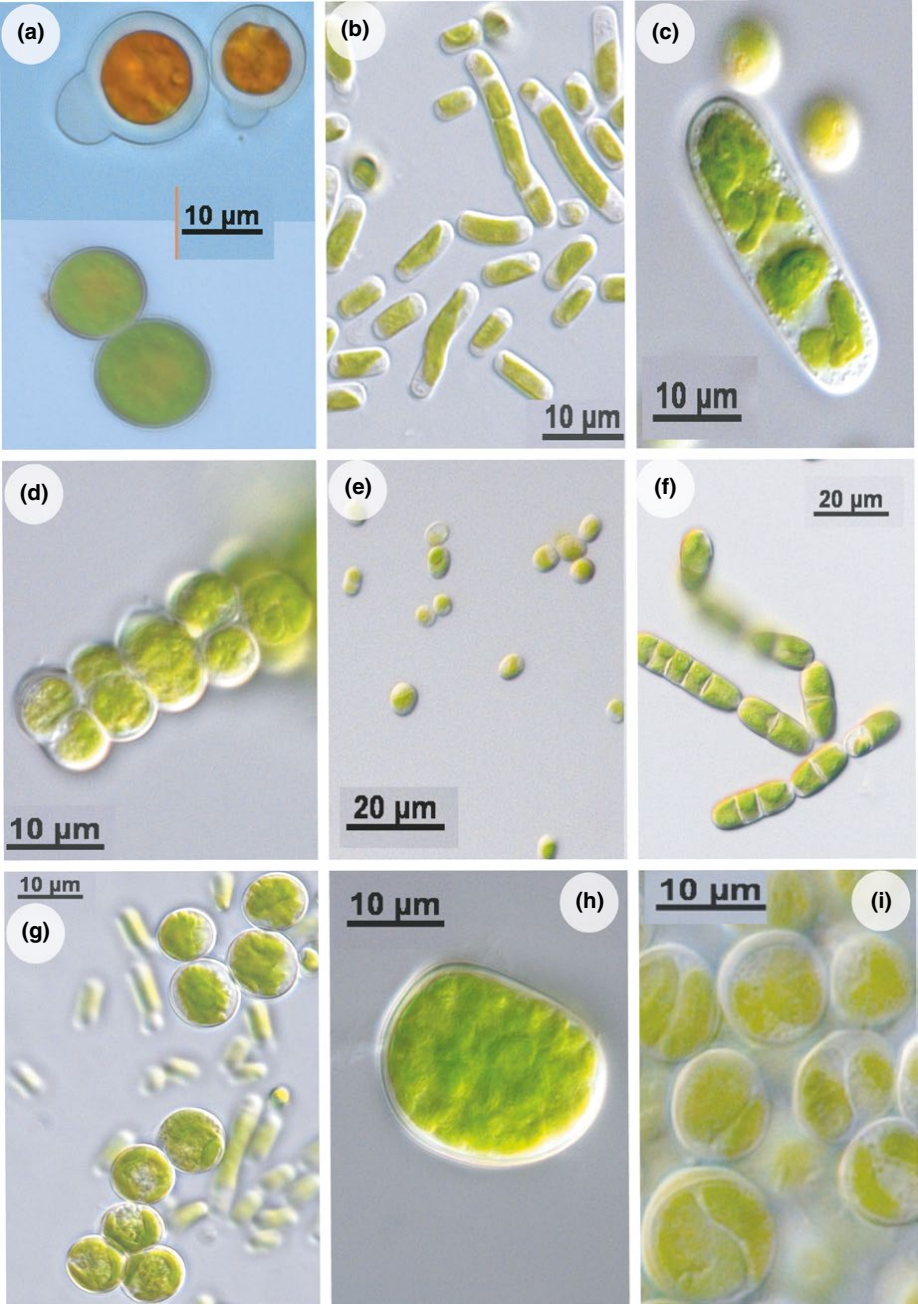
Free‐living algae identified in transplant vicinity (a) *Haematococcus* sp. (Flot. Emend. Wille) in a degenerated state and healthy state (b) *Stichococcus bacillaris* (Nägeli) (c) *Cylindrocystis brebissonii* (Meneghini) (d) *Chlorosarcinopsis* sp. (Herndon) (e) *Chlorella cf. miniata* ((Nägeli) Oltsmanns) (f) *Klebsormidium* sp. (Silva, Mattox et Blackwell) (g): *Trebouxia* sp. (Puymaly) (T) (h) *Myrmecia cf. biatorellae* (Tschermak‐Woess & Plessl) (i) *Myrmecia cf. irregularis* (J.B. Petersen)

Typical soil algae were identified from the cultured soil material (Figure 4), including free‐living *Trebouxia* and *Myrmecia* species. This suggests that photobiont pools reside in the transplant vicinities, which could potentially be available for the lichen to incorporate into developing thalli.

**Figure 5:**
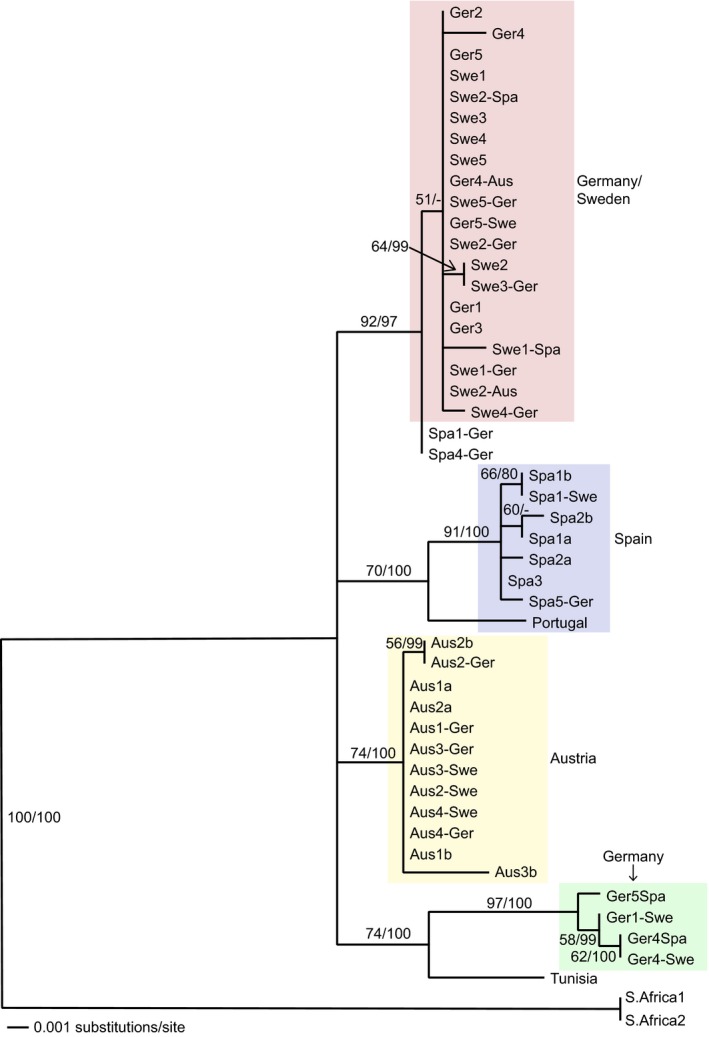
Maximum‐likelihood tree of *Psora decipiens* using data from the nuITS. Numbers at nodes represent first the ML bootstrap support (values ≥50%) and second the posterior probabilities from the Bayesian analysis (values ≥70%). Ger = Germany, Swe = Sweden, Spa = Spain, Aus = Austria. Examples: Ger2 = Germany replicate 2 control and Ger1– Aus = Germany replicate 1 transplanted to Austria

The following phase of the investigation was intended to ensure that the new‐grown thalli originated from the transplanted lichen rather than being native to the site. The fungal phylogeny was constructed from 49 sequences and demonstrates that the *P. decipiens* samples included in this study fall into four well‐supported clades (Figure 5). Two different genotypes appear to occur in Germany, one only includes transplants and the other is also the genotype found in Sweden. Therefore, it cannot be determined whether the new growth identified in transplants between Germany and Sweden originated from the transplanted material. Nevertheless, for the other transplants, except Spain1 to Germany (Spa1‐Ger) and Spain4 to Germany (Spa4‐Ger), it is clear that the thalli sequenced were transplant material. For example, the sample Austria3 that was transplanted to Sweden (Aus3‐Swe) falls within the Austria clade. The Spain1 to Germany and Spain4 to Germany transplants fall within the Germany/Sweden clade; this suggests that the new‐grown thalli did not originate from the transplant (Spain) but were of the host site (Germany). Therefore, only one replicate transplanted from Spain to Germany (Spa5–Ger) had true new‐grown thalli as the corresponding sequence falls within the Spanish clade.

**Figure 6:**
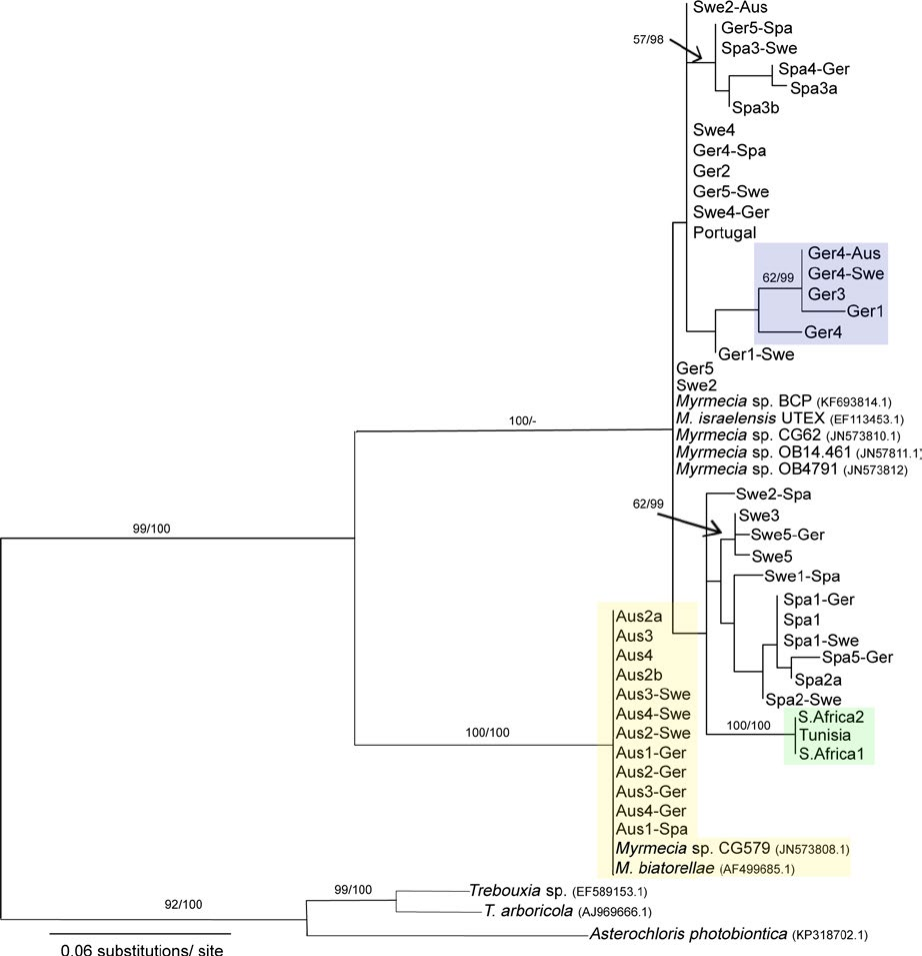
Maximum‐likelihood tree of *Psora decipiens* photobionts using data from the 26S rDNA and rbcL. Numbers at nodes represent first the ML bootstrap support (values ≥50%) and second the posterior probabilities from the Bayesian analysis (values ≥70%).Ger = Germany, Swe = Sweden, Spa = Spain, Aus = Austria. Example Swe2‐Aus = Sweden replicate 2 transplanted to Austria

The algal phylogeny was constructed in order to investigate whether photobiont switching takes place during a lichens acclimation process. Due to so many newly grown thalli appearing during the transplantation time, it was considered possible that the new thalli had incorporated a locally adapted alga as photobiont. The discovery of the disappearance of the photobiont layer in many of the samples, new growth and old, initially suggested that this did not take place. The molecular analysis corroborates this as transplant photobiont sequences belong to the original site. Although there are not clearly defined clades in the algal phylogeny as in the fungal, it is clear that the Austrian photobiont of *P. decipiens* is a single genotype which clusters together with *Myrmecia biatorellae* (accession number: AF499685.1) with 100% bootstrap and posterior probability values (Figure 6). In addition, in this experiment, the photobiont did not change when transplanted to a new site, even though in some cases it survived. In contrast, the analysis for Germany, Sweden, and Spain samples did not resolve any clear clades or gain high support. Sequence identity within this group, for both genes, was also found to be 99%. When the sequences from the Germany, Sweden, and Spain group were compared to the Austrian sequences, similarity was only found to be 94%; however, within the Austrian group, sequences are identical. The African samples, which grouped together and were included to increase levels of comparison, were found to be a distinct and separate group within the greater low‐altitude European clade. All sequences of the *Myrmecia* incorporated from GenBank*,* including *Myrmecia israelensis* (accession number: EF113453.1), that did not fall into the Austrian clade, were highly similar to each other (99%), and the Spain, Sweden, and Germany group.

## 4 Discussion

The purposes of this study were to investigate the acclimation potential of a lichen species that is found in climatically diverse BSC ecosystems. This would suggest that an important and widespread BSC species can successfully navigate environmental change. Although the lichen was found to survive and even produce new thalli when transplanted between sites, the algal layer was frequently found to have disappeared. Transplantation between sites, where an extreme climatic shift occurred, resulted in lichens unable to photosynthesise and therefore would be assumed to soon die. *Psora decipiens* has been shown to be composed of at least four genetically and geographically distinct groups, with a narrow range of algal symbionts that apparently cannot be switched when introduced into a new environment. The German and Sweden sites sharing a genotype highlights their similarities, they have been shown to be the most climatically similar, share the most lichen species, and have comparable BSC compositions (Büdel et al., 2014), including cyanobacterial assemblages (Williams et al., 2016). This lends supports to the suggestion that the genotypic and morpho‐physiological variations found within *P. decipiens* have a climatic determinant.

Austrian *P. decipiens* was found to have a distinct, single, algal genotype, clearly separate from the other sites; this led to the conclusion that photobiont switching did not take place. However, the lack of genetic diversity, low support, and deficiency of clades based on site, for the rest of the transplants, makes it impossible to completely disprove photobiont switching in this case. Nevertheless, it seems unlikely. Transplant and photobiont survival only occurred in sites that had similar or milder climatic conditions than the original. Allocation of resources has previously been shown to allow acclimation in lichens on a seasonal, moderate basis (Schofield, Campbell, Funk, & MacKenzie, 2003). The lichens surviving transplantation between Sweden and Germany is unsurprising considering the similar environmental conditions and a corresponding mycobiont genotype. Therefore, the transplants did not necessarily need to acclimate any further than what would already occur, on a seasonal basis, in their native habitat. Transplantation survival from an extreme to a mild site can also be explained by climatic conditions; an extreme site is not extreme all year around, and conditions at certain times would be very similar to those in a mild site, during which the lichens would be active, compared to harsh periods (high summer in Spain) where lichens are, for the most part, dormant (Raggio et al., 2014; Schroeter et al. 2010). However, it should be noted that this is grossly simplifying the effect of environmental conditions on lichens and does not take microclimate into consideration. In this case, transplants survived, died, or lost their algal layer and did not show a tendency to acclimate or to switch photobionts over 2 years. Increasing the time period may allow the effects of a milder climate on an extreme climate‐adapted sample to be further comprehended.

One surprising facet of this study was the transplantation samples' new growth being found to no longer have an algal layer. An explanation for the transplants being able to produce new thalli before environmental conditions became detrimental is due to the time of year the transplantation experiment was set up. The winter months in the arid Spanish site are when BSC organisms are active, with relatively little activity during the extreme summer months (Raggio et al., 2014). The transplants were installed in October 2012, during the growing season, 6–7 months before a harsh summer returned. A similar situation occurred in Austria, the samples were transplanted in July 2012, the middle of the alpine sites' growing season (Colesie, Green, Raggio, & Büdel, 2016). Consequently, it can be assumed that there was sufficient time for new growth before climatic conditions became detrimental to the transplanted lichens. It has been suggested that mycobionts may survive without a photobiont for up to a year (Etges & Ott, 2001) or 8 months when subjected to starvation stress (Zhang & Wei, 2011). However, it is generally believed that mycobionts must associate with an algal partner, even an incompatible one, within a short time period (Honegger, 1992). Therefore, it is possible that over more time a new photobiont could have been incorporated and the results show a potential pool of free‐living algae that could be utilized. It has been broadly suggested that lichens would switch their photobiont through a fungal spore associating with a new algal strain (Etges & Ott, 2001; Hedenås, Blomberg, & Ericson, 2007; Sanders & Lücking, 2002) or through taking algae from soredia (Ott, 1987), which are packets of fungi and algae that are easily dispersible and therefore frequently available. Additionally, lichens have also been known to steal a photobiont from other lichens during early stages of thallus development (Friedl, 1987; Lücking & Grube, 2002; Stenroos, 1990; Wedin et al., 2016). With these mechanisms in mind, it seems unlikely, although not impossible, that a fully formed thallus would be able to integrate a new alga, although a longer term transplantation experiment would be required to fully explore this.

**Figure A1:**
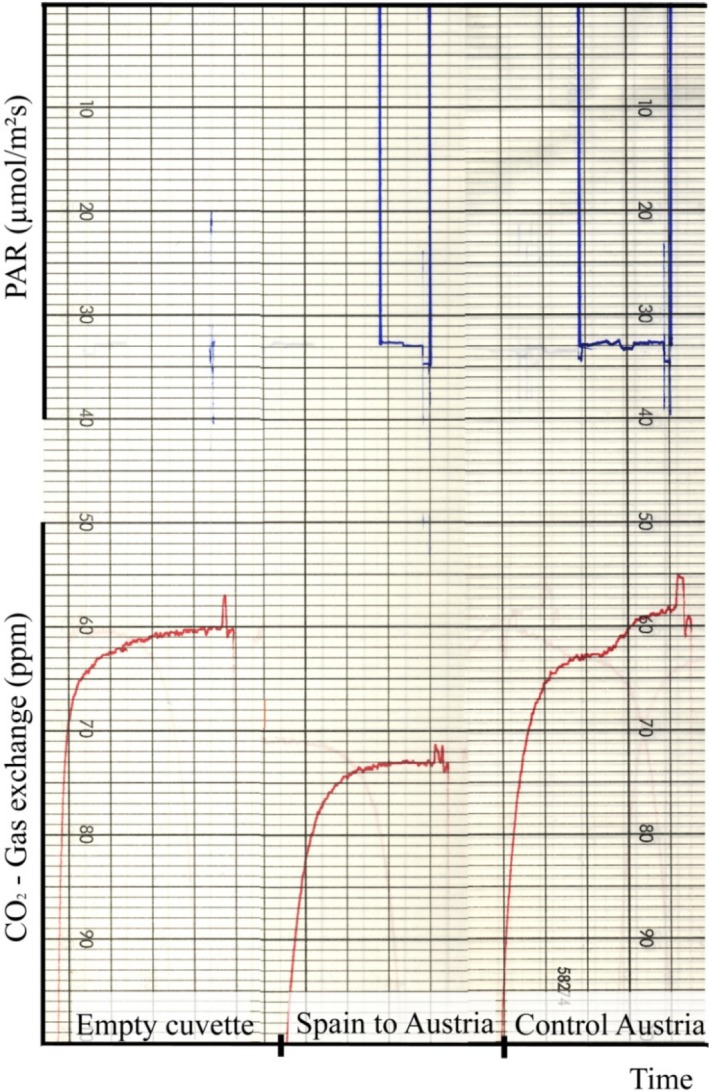
Gas exchange of *Psora decipiens* transplant, from Spain to Austria, and Austrian Control. Upper graph shows the emitted photosynthetic active radiation (PAR) and the lower the gas exchange response. The control sample shows release of CO_2_ (Photosynthesis) compared to the transplant, which had no remaining algal layer, which takes up CO_2_ (respires)

When lichens become stressed, respiration has been shown to increase by a significant margin (Kappen & Lange, 1972). A small‐scale physiological experiment on the transplanted lichens with no remaining algal layer showed extremely high levels of respiration, and as was expected, no photosynthetic activity (Figure A1 in Appendix). Taken together, the findings of site‐specific fungal genotypes, lack of differentiation between algal genotypes, and the SEM pictures showing the fungal hyphae growing within the algal cell cavities, without remaining algal cell material, suggest a hypothesis. The new climatic conditions initiated a stress response in the lichen, as can be determined by the high respiration levels. The increased respiration levels could not be sustained by the photosynthesising algae, and eventually, the fungi utilized the algae itself as a resource to sustain the high levels of respiration when active. This may be the reason for the algal cells having disappeared rather than just died and the fungal hyphae taking their place. Of course, this can only at this stage be speculation as no other studies have addressed this area of lichen biology.

Although the aim of this study was not to discuss photobiont diversity of *P. decpiens,* a finding which contradicts previous research has been an interesting outcome. *Psora decipiens* had previously been thought to associate with *Asterochloris* (Schaper & Ott, 2003) and/or *Trebouxia* photobionts, and also to be highly diverse (Ruprecht et al., 2014). However, the results presented here suggest that the green algal genus *Myrmecia* is the photobiont of all samples included in this study, and none of our photobionts grouped with *Trebouxia* or *Asterochloris*, as would have been expected from the literature. In addition, the actual diversity is surprisingly low, with only two clearly separate genotypes expressed. Indeed, this analysis is only based on two genes, the 26S rDNA and rbcL, and perhaps information from ITS regions would provide further delimitation between samples. However, a recent study by Škaloud, Steinová, Řídká, Vančurová, and Peksa (2015) convincingly shows high support for separate clades within the *Myrmecia* clade. One of the suggested clades contains the species *M. biatorella* J.B.Petersen and the other *M. israelensis* (S.Chantanachat & H.Bold) T.Friedl. This finding mimics very closely what was found in the analysis of the *P. decipiens* photobiont in this study. Previously, *Myrmecia* has been found to associate with a single lineage within the lichen family Verrucariaceae, which also identified only *M. biatorella* and *M. israelensis* species as photobionts (Thüs et al., 2011). These lichen species (*Placidium* sp. and *Heteroplacidium* sp.) are also associated with BSC, occur in the same habitats as *P. decipiens*, and share a similar morphology.

In conclusion, *Psora decipiens* may be considered a cosmopolitan soil crust lichen species; however, these results demonstrate that the species includes different genotypes that apparently cannot acclimate to changing environmental conditions within the species range. This is a small‐scale study considering that *P. decipiens* has a nearly worldwide distribution, and therefore to understand the genetic diversity and biogeography of this species, much larger scale studies are required. There is no evidence that photobiont switching takes place, and it currently seems that *P. decipiens* associates with a narrow range of photobionts within the small genus *Myrmecia*. Due to the contradicting research, and limited sampling sites, regarding the photobiont of this species, further research is undeniably required. Continued investigation is necessary to further answer questions about the ability of lichens to acclimate; however, it is currently clear that climate change and habitat loss may be severely detrimental to the continued survival of this important lichen species.

## Author Contribution

Laura Williams (first author) designed, implemented, analyzed, interpreted, and prepared the manuscript for this research. Claudia Colesie contributed to all aspects of the research and writing, implemented the SEM, and provided essential ideas and interpretation of results. Anna Ullmann conducted initial investigation on samples after transplantation and helped standardize the methodology used subsequently. Martin Westberg key participant in investigating the genetic diversity of *Psora decipiens,* assisted in implementing the research design, and provided support throughout data interpretation and manuscript preparation. Mats Wedin assisted with research design and implementation including essential guidance and support with acquisition of permits. Provided support throughout the research project and assisted in manuscript editing. Burkhard Büdel SCIN project principal investigator and recipient of DFG grant. Provided knowledge and support throughout the research project and contributed to construction and editing of manuscript.

## Conflict of Interest

None declared.

## Appendix 1

### 1.1

**Table A1:** *Psora decipiens* samples used in the present study and 26S and rbcL accession numbers for algal and fungal sequences (European Nucleotide Archive: www.ebi.ac.uk/ena/data/view/LT717490-LT717626)

Specimen	Origin	Transplanted to	26S algae	rbcL algae	26S fungi
Swe1	Öland, Sweden	–	–	–	LT717490
Swe2	Öland, Sweden	–	LT717573	LT717610	LT717491
Swe3	Öland, Sweden	–	LT717574	LT717611	LT717522
Swe4	Öland, Sweden	–	LT717576	LT717613	LT717492
Swe5	Öland, Sweden	–	LT717578	LT717615	LT717493
Swe1‐Ger	Öland, Sweden	Gössenheim, Germany	–	–	LT717517
Swe2‐Ger	Öland, Sweden	Gössenheim, Germany	–	–	LT717519
Swe3‐Ger	Öland, Sweden	Gössenheim, Germany	–	–	LT717521
Swe4‐Ger	Öland, Sweden	Gössenheim, Germany	LT717575	LT717612	LT717523
Swe5‐Ger	Öland, Sweden	Gössenheim, Germany	LT717577	LT717614	LT717524
Swe1‐Spa	Öland, Sweden	Almeria, Spain	LT717570	LT717607	LT717516
Swe2‐Spa	Öland, Sweden	Almeria, Spain	LT717571	LT717608	LT717518
Swe2‐Aus	Öland, Sweden	Hochtor, Austria	LT717572	LT717609	LT717520
Ger1	Gössenheim, Germany	–	LT717550	LT717590	LT717499
Ger2	Gössenheim, Germany	–	LT717551	LT717591	LT717506
Ger3	Gössenheim, Germany	–	LT717552	LT717592	LT717500
Ger4	Gössenheim, Germany	–	LT717555	LT717596	LT717507
Ger5	Gössenheim, Germany	–	LT717558	LT717599	LT717508
Ger1‐Swe	Gössenheim, Germany	Öland, Sweden	LT717549	LT717589	LT717498
Ger4‐Swe	Gössenheim, Germany	Öland, Sweden	LT717554	LT717595	LT717503
Ger5‐Swe	Gössenheim, Germany	Öland, Sweden	LT717557	LT717598	LT717505
Ger4‐Spa	Gössenheim, Germany	Almeria, Spain	LT717553	LT717593	LT717501
Ger5‐Spa	Gössenheim, Germany	Almeria, Spain	LT717556	LT717597	LT717504
Ger4‐Aus	Gössenheim, Germany	Hochtor, Austria	–	LT717594	LT717502
Aus1a	Hochtor, Austria	–	–	–	LT717533
Aus1b	Hochtor, Austria	–	–	–	LT717525
Aus2a	Hochtor, Austria	–	LT717569	LT717621	LT717534
Aus2b	Hochtor, Austria	–	LT717559	LT717616	LT717527
Aus3	Hochtor, Austria	–	LT717560	LT717622	LT717529
Aus4	Hochtor, Austria	–	LT717561	LT717623	–
Aus2‐Swe	Hochtor, Austria	Öland, Sweden	LT717564	LT717602	LT717511
Aus3‐Swe	Hochtor, Austria	Öland, Sweden	LT717566	LT717604	LT717513
Aus4‐Swe	Hochtor, Austria	Öland, Sweden	LT717568	LT717606	LT717515
Aus1‐Ger	Hochtor, Austria	Gössenheim, Germany	LT717562	LT717600	LT717509
Aus2‐Ger	Hochtor, Austria	Gössenheim, Germany	LT717563	LT717601	LT717510
Aus3‐Ger	Hochtor, Austria	Gössenheim, Germany	LT717565	LT717603	LT717512
Aus4‐Ger	Hochtor, Austria	Gössenheim, Germany	LT717567	LT717605	LT717514
Spa1a	Almeria, Spain	–	LT717541	LT717618	LT717530
Spa1b	Almeria, Spain	–	–	–	LT717526
Spa2a	Almeria, Spain	–	LT717539	LT717619	LT717531
Spa2b	Almeria, Spain	–	–	–	LT717528
Spa3	Almeria, Spain	–	LT717540	LT717620	LT717532
Spa3b	Almeria, Spain	–	LT717542	LT717617	–
Spa1‐Swe	Almeria, Spain	Öland, Sweden	LT717544	LT717584	LT717495
Spa2‐Swe	Almeria, Spain	Öland, Sweden	LT717545	LT717585	–
Spa3‐Swe	Almeria, Spain	Öland, Sweden	LT717546	LT717586	–
Spa1‐Ger^a^	Almeria, Spain	Gössenheim, Germany	LT717543	LT717583	LT717494
Spa4‐Ger^a^	Almeria, Spain	Gössenheim, Germany	LT717547	LT717587	LT717496
Spa5‐Ger	Almeria, Spain	Gössenheim, Germany	LT717548	LT717588	LT717497
Portugal	Portugal	–	LT717579	LT717624	LT717535
Tunisia	Tunisia	–	LT717582	LT717626	LT717538
S.Africa1	South Africa	–	LT717580	–	LT717536
S.Africa2	South Africa	–	LT717581	LT717625	LT717537

a Fungal phylogeny demonstrates that identified new growth did not originate from Spanish sample.
