# Corrigendum

**DOI:** 10.1002/ece3.3320

**Published:** 2017-09-06

Williams, L., Colesie, C., Ullmann, A., Westberg, M., Wedin, M., & Büdel, B. (2017) Lichen acclimation to changing environments: Photobiont switching vs. climate‐specific uniqueness in *Psora decipiens. Ecology and Evolution, 7,* 2560‐2574.

Figure A1. The CO_2_ exchange as ΔCO_2_ between sample and reference gas stream. The empty cuvette values are used as a reference for no gas exchange. Values above this reference line indicate CO_2_ uptake (photosynthesis), and values below this reference indicate a CO_2 _release (respiration). The transplanted samples show a strong respiration with no response to light, indicating that no photosensitive reaction is possible, as no photobiont layer is remaining. The Austrian control shows respiration in darkness, and as soon as light is applied, the signal crosses the reference line and net photosynthesis is positive.

